# Magnetic Mg-Fe/LDH Intercalated Activated Carbon Composites for Nitrate and Phosphate Removal from Wastewater: Insight into Behavior and Mechanisms

**DOI:** 10.3390/nano10071361

**Published:** 2020-07-12

**Authors:** Omar Alagha, Mohammad Saood Manzar, Mukarram Zubair, Ismail Anil, Nuhu Dalhat Mu’azu, Aleem Qureshi

**Affiliations:** Environmental Engineering Department, College of Engineering A13, Imam Abdulrahman Bin Faisal University, Main Campus, P.O. Box 1982, Dammam 34212, Saudi Arabia; msmanzar@iau.edu.sa (M.S.M.); mzzubair@iau.edu.sa (M.Z.); ianil@iau.edu.sa (I.A.); nmdalhat@iau.edu.sa (N.D.M.); aqureshi@iau.edu.sa (A.Q.)

**Keywords:** waste sludge-based activated carbon, magnetic layered double hydroxide, phosphate and nitrate removal, wastewater treatment, regeneration potential

## Abstract

This experimental work focused on the synthesis, characterization, and testing of a unique, magnetically separable, and eco-friendly adsorbent composite material for the advanced treatment and efficient removal of nitrate and phosphate pollutants from wastewater. The MgAl-augmented double-layered hydroxide (Mg-Fe/LDH) intercalated with sludge-based activated carbon (SBAC-MgFe) composites were characterized by FT-IR, XRD, BET, VSM, SEM, and TEM techniques, revealing homogeneous and efficient dispersion of MgFe/LDH within the activated carbon (AC) matrix, a highly mesoporous structure, and superparamagnetic characteristics. The initial solution pH, adsorbent dose, contact time, and temperature parameters were optimized in order to reach the best removal performance for both pollutants. The maximum adsorption capacities of phosphate and nitrate were found to be 110 and 54.5 mg/g, respectively. The competition between phosphate and coexisting ions (Cl^−^, CO_3_^2−^, and SO_4_^2−^) was studied and found to be remarkably lower in comparison with the nitrate adsorption. The adsorption mechanisms were elucidated by kinetic, isotherm, thermodynamic modeling, and post-adsorption characterizations of the composite. Modeling and mechanistic studies demonstrated that physisorption processes such as electrostatic attraction and ion exchange mainly governed the nitrate and phosphate adsorption. The composite indicated an outstanding regeneration performance even after five sequences of adsorption/desorption cycles. The fabricated composite with magnetically separable characteristics can be used as a promising adsorbent for the removal of phosphate and nitrate pollutants from wastewater.

## 1. Introduction

Water is an invaluable commodity that needs to be preserved and stored appropriately. The majority of the Earth’s surface is enclosed by water. An insignificant percentage of fresh water, which is only 0.6%, is used as drinking water. Population growth, industrial development, urbanization, and agricultural activities have given rise to a higher level of contaminants. The persistently rising percentage of contaminants degrades the quality of the water. Nitrate and phosphate finally enter the hydrosphere from the fertilizer or chemical industries, which also makes water unsuitable for drinking or other uses. Consequently, water resources must be accurately managed, and the treatment of wastewater must be proficiently performed.

Phosphate and nitrate are the main supplements required by corporeal microbes for their biological development. These nutrients, when present in disproportionate quantities, contaminate lagoons, watercourses, and marshlands [1]. The wide-ranging sources of additional nitrate, nitrite, and phosphate come from built structures, animal fodder, pastoral manures, industrialized left-overs, and trash scrapyards [2]. Phosphorus release from anthropogenic activities is considered to be the main reason for unbalanced algae development and ruined stream water quality [3]. As a result, there is a vital need to plan an effectively and profitably designed wastewater treatment system to remove excessive nitrate and phosphate ions before releasing them into the land site or else for re-use.

Biochar is charcoal generated from plant stock and kept in topsoil as a means of eliminating carbon dioxide from the air; it has been of noteworthy interest for use in soil enrichment and the elimination of noxious contaminants from water bodies. Biochar is permeable in nature, which permits it to hold water and other nutrients dissolved in the water [4]. Several studies have shown that biochar is a desirable and efficient adsorbent, owing to its exceptional physical features, adequate surface flexibility, and excellent ion exchange capability [5,6]. The permeable pattern of biochar has an extraordinary specific surface area, which displays enhanced uptake of several contaminants from the aqueous medium [7,8,9].

Layered double hydroxides (LDHs), which are correspondingly acknowledged as hydrotalcites, have recently drawn significant attention, and they have the potential to play a role in many schemes like cation exchangers, catalysts, and the treatment of organic, inorganic and anionic contaminants [10]. Gupta et al. [11] developed a Zn-Fe/LDH by using the coprecipitation method and determined satisfactory phosphate sorption to be 36 mg/g. Santos et al. [12] established moderate adsorption performance of nitrate on an Mg-Fe-Cl/LDH. Liu et al. [13] prepared very thin Mg-Al/LDH nanoparticles by the fast one-step synthesis of 3–5 nm thick LDH nanoparticles, using urea as a diffusing agent, and indicated a maximum sorption capacity of 98 mg/g.

Currently, numerous research works have been involved in developing biochar and other low-cost materials from waste products as excellent adsorbents to boost the remediation of aqueous pollutants [8,14,15]. For instance, Chen et al. [16] revealed that a high nitrate removal rate (706 mg/L) could be achieved by a methane-based membrane biofilm reactor. Similarly, Ivanets et al. [17] reported that thermally treated (800 °C) dolomite could effectively bind 0.75 to 3.0 mmol/g of phosphorus. Interestingly, the deposition of LDH on the biochar matrix was revealed as a favorable method to substantially improve the resultant material properties, showing remarkable removal performance for water contaminants due to the synergistic effect of biochar and LDH [18]. Tran et al. [14] inspected modified Mg-Al/LDHs derived from tobacco stalk and reported a better phosphate adsorption capability relative to other biochars, with an adsorption capability of 41 mg/g. In 2016, Xue et al. [19] developed Mg-Fe/LDH particles carrying wheat-straw biochar and revealed their excellent ability to remove nitrate from water. Similarly, Yin et al. [20] inspected Mg-Al-modified biochar to eliminate nitrate and phosphate ions from nutrient-rich water and showed an outstanding affinity, with a maximum sorption capability of 41 mg/g for nitrate ions and 74 mg/g for phosphate ions. In another study, You et al. [21] validated Fe-Al-modified biochar as an effective adsorbent for the removal of phosphate from wastewater. In the previously published study of our research group, a date palm-derived biochar intercalated with Mg-Al/LDH composites showed enhanced sorption capacities of nitrate and phosphate ions from a water solution; 178 and 28 mg/g, respectively [22]. Based on an extensive literature survey, Mg-Fe/LDH composites intercalated with biochar derived from sewage sludge has not yet been inspected for the removal of nitrate and phosphate ions in both simulated and actual wastewaters.

Therefore, the main objective of this research work is to efficiently treat nitrate and phosphate ions in wastewaters using a new, eco-friendly, and sustainable material: a magnetic Mg-Fe/LDH intercalated with activated carbon (AC) derived from waste sludge at different mass ratios. The physiochemical characteristics of the fabricated sludge-based activated carbon (SBAC-MgFe) composites were comprehensively analyzed using various characterization techniques, including FT-IR, XRD, BET, VSM, SEM, and TEM. The effective factors involved in the adsorption mechanisms of nitrate and phosphate ions, such as temperature, initial solution pH, composite dosage, coexisting ions, and adsorption time, were also investigated and optimized. Phosphate and nitrate mechanistic studies were thoroughly evaluated, considering isotherm, kinetic, thermodynamic modeling studies, and the characterization of composites after phosphate and nitrate adsorption. Following these analyses, the reusability of the composite material was tested by regeneration performance studies, and the nitrate and phosphate removal performances of the material were investigated in real wastewater samples.

## 2. Materials and Methodology

### 2.1. Chemicals

Waste sludge-based ZnCl_2_ AC was manufactured in accordance with our previous work, utilizing the waste sludge of a domestic wastewater treatment plant using an activated sludge process in Dhahran city, Western Region of Saudi Arabia [23]. All chemicals were purchased from Sigma Aldrich Co. (USA), and were of analytical grade (purity 99.99%). The materials utilized were composed of iron(III) nitrate nonahydrate (Fe(NO_3_)_3_·9H_2_O), magnesium nitrate hexahydrate (Mg(NO_3_)_2_·6H_2_O), potassium di-hydrogen phosphate (KH_2_PO_4_), sodium nitrate (NaNO_3_), sodium phosphate (Na_3_PO_4_), sodium sulfate (Na_2_SO_4_), sodium carbonate (Na_2_CO_3_), sodium chloride (NaCl), hydrochloric acid (HCl), and sodium hydroxide (NaOH).

### 2.2. Synthesis of Magnetic Mg-Fe/LDH Composites

**Scheme 1:**
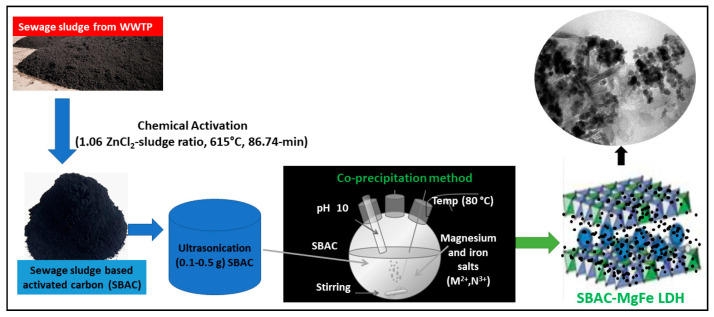
Schematic diagram of the synthesis of sludge-based activated carbon (SBAC-MgFe) composites via coprecipitation method.

The coprecipitation method was used to prepare sewage sludge-based activated carbon (SBAC) incorporated with magnesium-iron layered double hydroxide (Mg-Fe/LDH) (SBAC-MgFe) composites. As shown in Scheme 1, initially, a known amount (100 mg and 500 mg) of ZnCl_2_ sewage sludge-based activated carbon (SBAC), prepared in a previous study [23], was homogeneously dispersed in 50 mL double distilled water via ultra-sonication for about 30 min. Concurrently, 0.01 M of iron(III) nitrate nonahydrate (4.04 g) and 0.01 M of magnesium nitrate hexahydrate (2.56 g) salts were mixed with ultrapure water (100 mL) in a round bottom flask. The metal ions and SBAC solution were admixed and then vigorously agitated at 60 °C for 15 min. Furthermore, the pH (10 ± 0.5) of the mixture was maintained by the dropwise addition of a freshly prepared solution of sodium hydroxide (0.1 M). After attaining the chosen pH, the reactor was refluxed for 18 h at 90 °C. The synthesized composite was washed with ultrapure water, followed by cleaning with analytical grade ethanol to remove any unreacted chemicals. The produced composite was further dehydrated in an oven for a period of 48 h at 85 °C. The SBAC-MgFe composite powder was then placed in a desiccator until the adsorption tests. Table 1 lists the design compositions of the SBAC-MgFe composites.

### 2.3. Characterization Methods for Magnetic SBAC-MgFe Composites

The SBAC-MgFe composites were thoroughly characterized by various kinds of techniques, including (i) X-ray diffraction (XRD) at a wavelength of 0.1542 nm, within 2θ range of 20° to 70° (0.02° per step) with an exposition time of 2 min per step (D8-advance XRD, Bruker™, MA, USA); (ii) Fourier transform infrared spectroscopy (FT-IR), in the range of 500 to 4000/cm at 32 running scans, with a resolution of 4/cm (Nicolet-6700, Thermo-Fisher-Scientific™, MA, USA); (iii) scanning electron microscopy (SEM) at a resolution of 10 μm to 100 nm at 20 kV, with a sample gold sputtering thickness of 5 nm (SM/LV/6460, JEOL™, MA, USA); (iv) Brunauer/Emmett/Teller (BET) and pore size distribution using multi-point BET and Barrett–Joyner–Halenda (BJH) method with N_2_ adsorption–desorption isotherm (25 points) (Series-Tristar-II, Micromeritics™, GA, USA). The magnetic properties of the samples were identified using a vibrating sample magnetometer (VSM) with an applied field strength of ±20 kOe at room temperature (Series-7400, LakeShore Cryotronics™, OH, USA).

### 2.4. Sorption Methodology for the Removal of Nitrate and Phosphate Ions

Batch adsorption experiments were performed using SBAC-MgFe composites to investigate the adsorption capacity of nitrate and phosphate. The adsorption experiments were conducted on a selected composite showing a higher removal performance in order to further investigate the effects of adsorption factors such as initial solution pH (3–12), composite amount (2–20) mg, coexisting ions (Cl^−^, CO_3_^2−^, and SO_4_^2−^) (0.001–0.1) M, contact time (0–360) minutes, temperature of (298–318) K, and stirring rate of 250 rpm. KH_2_PO_4_ and NaNO_3_ were used as synthetic sources of phosphate and nitrate ions, respectively. Standard solutions of phosphate and nitrate with 1000 mg/L concentrations were prepared using ultrapure water obtained from the Milli-Q^®^ direct water purification system (18.2 MΩ·cm resistivity). The concentration of nitrate and phosphate ions was quantified using an ultraviolet/visible (UV/visible) spectrophotometer (DR/6000 Benchtop, Hach, Colorado/USA). The adjustment in pH to the primary favored value was attained by the use of 0.1 M HCl and NaOH solutions. The adsorption experiments were performed in triplicate, and their average values are demonstrated in the following sections. The removal efficiencies (*RE, %*) and the sorption capacities (*q_e_*) were assessed by employing Equations (1) and (2).

(1) $$RE\;\left(\%\right)=\frac{C_{0}-C_{e}}{C_{0}}\times 100$$

(2) $$q_{e}=(C_{0}-C_{e})\;\frac{V}{m}$$

where, *C*_0_ and *C_e_* stand for the initial and at equilibrium concentrations (as mg/L) of phosphate and nitrate anions separately; *q_e_* stands for the adsorption capacity of nitrate and phosphate anions adsorbed (as mg) for a unit mass of the adsorbent (mg/g) at equilibrium condition; *V* is the volume of the liquid medium (L); *m* stands for the dose of the sorbent in the liquid medium (g).

### 2.5. The Effects of Coexisting Ions

Actual wastewater contains different anions, such as Cl^−^, NO_3_^−^, CO_3_^2−^, SO_4_^2−^, and PO_4_^3−^, that might influence the removal of nitrates or phosphates in the adsorption process. Consequently, NaCl, NaNO_3_, Na_2_CO_3_, Na_2_SO_4_, and Na_3_PO_4_ salts (ACS reagent grade, ≥99.0%) were prepared within a concentration range of 0.001 M to 0.1 M to study the effects of coexisting ions on the removal efficiencies of nitrate and phosphate. The competitive ion studies were performed at a 40-mL solution volume containing a target anion (10 mg/L) with a coexisting anion (0.001, 0.01, and 0.1 mol/L).

### 2.6. Equilibrium, Isotherm, and Kinetic Studies

Equilibrium, isotherm, and kinetic studies were performed at nitrate and phosphate concentrations in the range of 10 to 50 mg/L and interaction time within 10–360 min at the levels of factors: (i) sorbent amount (5 mg); (ii) pH (3 and 6); (iii) stirring rate (250 rpm); and (iv) temperature (298–318) K. The initial and equilibrium concentrations of nitrates and phosphates were quantified by the Hach^®^ TNT-plus-TM barcoded test kits on the DR/6000 UV-Visible spectrophotometer. The nitrate and phosphate anion sorption process on the magnetic SBAC-MgFe composites was validated by the use of kinetic and isotherm models, as well as the characterization of the composites after the adsorption of nitrates and phosphates. Consequently, in the present study, kinetic models, including pseudo 1st, 2nd order, film, and intraparticle diffusion models [24], were used, whereas the Langmuir [25] and Freundlich [26] isotherm models were employed in the equilibrium investigation studies.

### 2.7. Sequenced Adsorption/Regeneration Experiments

The adsorption/regeneration experimentation was performed at 10 mg/L concentrations of both nitrate and phosphate ions in a 150-mL solution volume, stirred at 250 rpm for 24 h. The adsorbent dosage was selected to be 50 mg for sequential experiments. After adsorption, the spent composite was recovered by applying 5 min centrifugation at a 5000 rpm rate. The spent composites were regenerated by the use of 0.1 M and 1.0 M concentrations of basic NaOH solutions. The regeneration of the produced SBAC-MgFe composites was evaluated by performing five consecutive adsorption/regeneration runs. Each adsorption/regeneration cycle was repeated in triplicate, and the average values are assessed in the Results section.

## 3. Results and Discussion

### 3.1. Characterization of Magnetic SBAC-MgFe Composites

**Figure 1:**
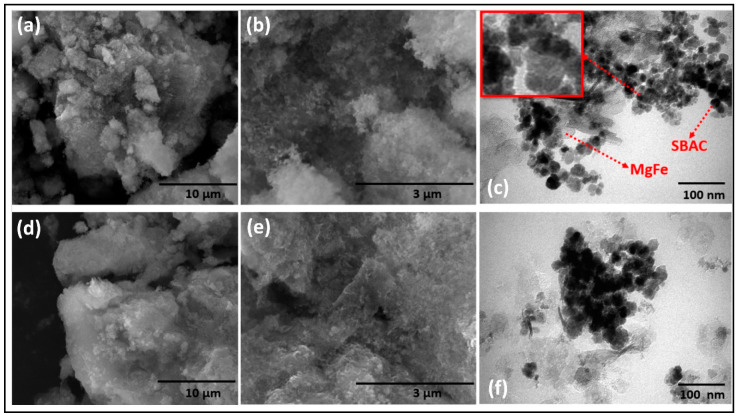
SEM images of SBAC_100_MgFe (**a**,**b**) and SBAC_500_MgFe (**d**,**e**) composites. TEM images of SBAC_100_MgFe (**c**) and SBAC_500_MgFe (**f**) composites.

The actual composition of the SBAC-MgFe composites, estimated using an energy dispersive X-ray (EDX) analysis, are indicated in Figure S1. The percentage distribution of major elements (C, O, Mg, Fe, and Si) in SBAC_100_MgFe and SBAC_500_MgFe composites was found to be (12.6%, 34.8%, 13.3%, 55.3%, and 1.5%) and (20.6%, 40.1%, 8.1%, 39.1%, and 1.6%), respectively. The surface structure and intercalation efficiency of SBAC within the segments of MgFe/LDH were assessed by SEM and TEM analyses, respectively. As shown in Figure 1a,b, the SBAC_100_MgFe composite exhibited a porous and rough surface structure surrounded by tiny particles of SBAC. Similarly, the presence of discrete patterns and the amorphous surface morphology of the SBAC_500_MgFe composite are attributed to a higher content (0.5 g) of amorphous SBAC (Figure 1d,e). TEM analysis of the SBAC-MgFe composites was performed to demonstrate further the interaction behavior of SBAC nanoparticles loading within the layers of MgFe/LDH. The TEM image of the SBAC_100_MgFe composite (Figure 1c) showed the formation of a hexagonal structure of MgFe/LDH, and SBAC particles were randomly dispersed within the surface of MgFe/LDH, indicating the heterogeneous surface morphology of the composite. The coprecipitation synthesis method and the appropriate content of SBAC (0.1 g) facilitate the better and more effective intercalation of SBAC into MgFe/LDH layers. This resulted in the improved surface, structure, and textural characteristics of the SBAC_100_MgFe composite, as confirmed by the FT-IR, XRD, and BET analyses discussed below.

However, as shown in Figure 1f, the presence of a high loading (0.5 g) of SBAC nanoparticles in the SBAC_500_MgFe composite caused undesired agglomerations into layers of MgFe/LDH, resulting in poor crystallinity and weak surface-active functional groups, as elucidated via the XRD and FT-IR analyses. Similar behavior has been reported by previous studies when synthesizing biochar LDH composites of different mass ratios [27,28].

**Figure 2:**
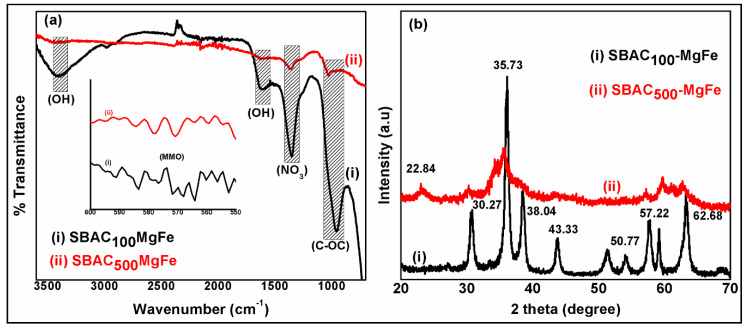
FT-IR spectra (**a**) and XRD patterns (**b**) of SBAC_100_MgFe and SBAC_500_MgFe composites.

Figure 2a shows the FT-IR spectra of the SBAC-MgFe composites. In the SBAC_100_MgFe composite spectra, the broad band recorded at 3444 cm^−1^ represents stretching type vibrations of (O-H) groups due to the occurrence of water molecules and OH bonding in the upper and MgFe/LDH interlayers [29,30]. The strong and sharp band observed at 1357 cm^−1^ in the SBAC_100_MgFe spectra corresponds to the nitrate anions (NO_3_^2−^) within the interlayers of the MgFe/LDH composites [31]. The existence of bands at 571 cm^−1^ might be associated with mixed metal oxides (Mg-O or Fe-O or MgFe-O). Moreover, the characteristic bands of SBAC_100_MgFe correspond to the C=O and C-O-C groups that are observed at 1609 and 953 cm^−1^, respectively. The presence of MgFe characteristic bands in the SBAC-MgFe composites indicates the efficient formation of LDH structures on the SBAC matrix. Besides this, SBAC_100_MgFe indicated stronger characteristic bands of both SBAC and MgFe/LDH compared to those of the SBAC_500_MgFe composite. This could be associated with the presence of a lower content of SBAC (0.1 g) in the SBAC_100_MgFe composite relative to 0.5 g of SBAC in the SBAC_500_MgFe composite, which resulted in better intercalation of SBAC within the layers of MgFe, leading to (i) a significant improvement in functional groups and (ii) the enhanced removal of anionic pollutants from wastewaters.

Figure 2b displays the X-ray diffraction patterns of the SBAC-MgFe composites. In the SBAC_100_MgFe pattern, sharp diffraction bands at two (theta) 30.27°, 35.73°, 38.04°, 43.33°, 57.22°, and 62.68° are attributed to (009), (012), (015), (018), (110), and (113) indexed planes, which are very similar to the MgFe/LDH nanoparticles reported by Durrani et al. [32]. The size of the crystallites of SBAC_100_MgFe calculated using the Scherrer formula is 30 nm, which is bigger than that of MgFe nanoparticles [33]. Besides this, the diffraction band which appeared at 22.84° in the XRD pattern of the SBAC_500_MgFe composite was attributed to the plane (002) of graphitic carbon [8]. The XRD results indicate that the SBAC_100_MgFe composite showed excellent crystallinity compared to the SBAC_500_MgFe composite. This could be ascribed to the excellent formation and efficient dispersion of MgFe nanoparticles within the AC matrix without comprising the crystallographic structure. In contrast, the poor crystalline structure of the SBAC_500_MgFe composite is associated with the disorder and lattice defects of the MgFe crystalline structure due to the high loading of SBAC, which agglomerates or is stacked into LDH segments [18,34].

**Figure 3:**
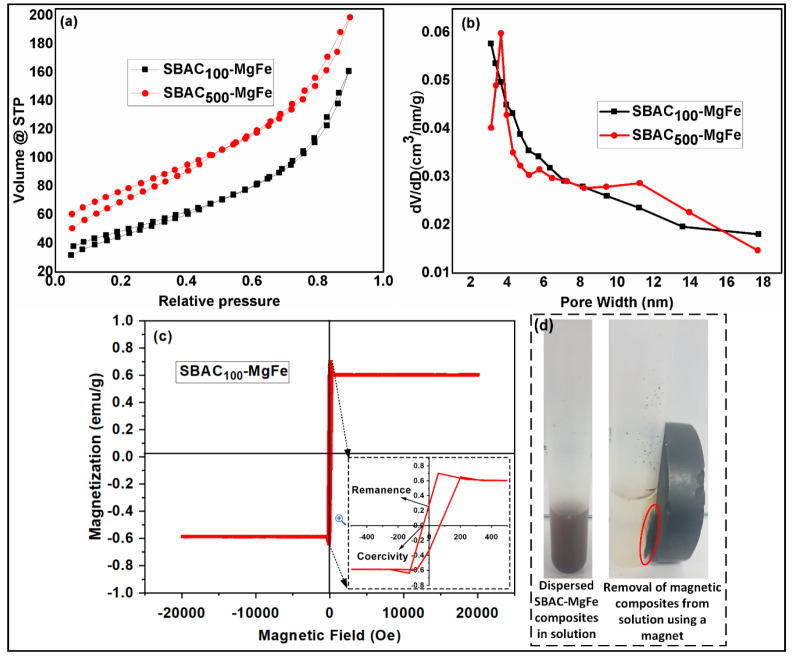
N_2_ adsorption–desorption isotherm curves for SBAC_100_MgFe and SBAC_500_MgFe composites (**a**); pore size distributions of SBAC_100_MgFe and SBAC_500_MgFe composites (**b**); vibrating sample magnetometry plot of SBAC_100_MgFe composite (**c**); removal of magnetic composites from solution using a magnet (**d**).

The surface and pore characteristics of the SBAC-MgFe composites, such as surface area, average pore volume, pore radius, and pore size distribution (BJH), were estimated by performing N_2_ adsorption–desorption isotherms. The N_2_ adsorption–desorption curves and pore size distribution plots of SBAC_100_MgFe and SBAC_500_MgFe composites are depicted in Figure 3a,b, and their textural results are listed in Table 2. For both SBAC-MgFe composites, the shape of the adsorption–desorption isotherm (Figure 3a) is similar to the hysteresis loops of Type IV, indicating that both composites are highly mesoporous. Accordingly, a higher fraction of pore volume observed at a diameter range 2.9–4.5 nm, according to the BJH pore size distribution results (Figure 3b), further confirms the mesoporous characteristics of the composites. The surface areas of SBAC_100_MgFe and SBAC_500_MgFe were found to be 169 and 257 m^2^/g, respectively, which are relatively higher than those of previously reported MgFe/LDH composites [35,36]. These results demonstrated that the incorporation of a small proportion of SBAC into MgFe/LDH segments is an effective and sustainable technique to produce composites with a high surface area, which may result in better uptake of anions via the surface adsorption mechanism.

The vibrating sample magnetometry (VSM) technique was applied to measure the magnetism (magnetic field dependent variation) of SBAC_100_MgFe, and the results are displayed in Figure 3c. The saturation magnetization of the SBAC_100_MgFe composite was 0.6–0.7 emu/g, which is almost ten times lower than the pristine MgFe/LDH [37]. This might be attributed to the formation of a low amount of MgFe_2_O_4_ spinel ferrites in the SBAC_100_MgFe LDH composite. Besides this, the magnetic remanence of SBAC_100_MgFe is nearly zero (0.26) and exhibits low coercivity (Figure 3c). This indicates that SBAC_100_MgFe is a soft ferromagnetic material and can be easily recovered from the liquid phase using an applied magnetic field, as displayed in Figure 3d.

### 3.2. Effects of pH, Adsorbent Dose, Contact Time, and Coexisting Ions on Adsorption

#### 3.2.1. Initial Solution pH

**Figure 4:**
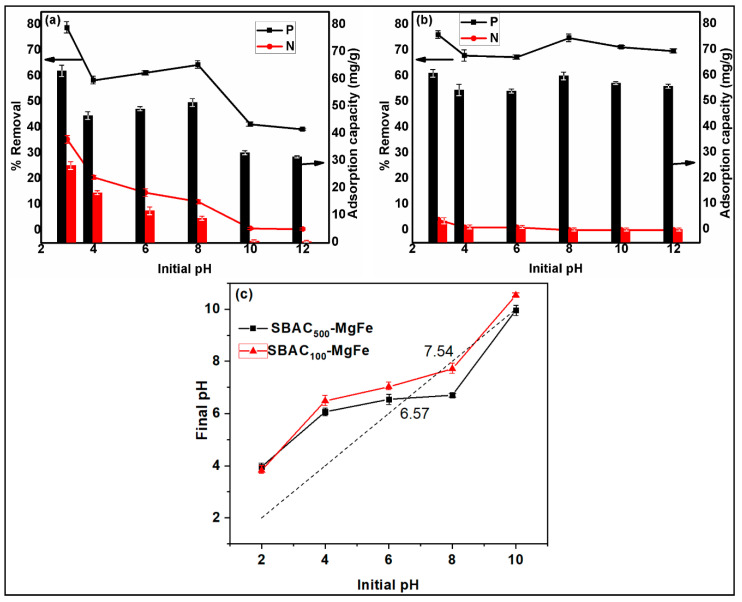
Effect of initial pH on the adsorption of SBAC_100_MgFe composite (**a**) and SBAC_500_MgFe composite (**b**); the point of zero charge plots for the SBAC_100_MgFe and SBAC_500_MgFe composites (**c**).

The initial solution pH plays a critical role in the surface characteristics of the sorbent, point of zero charge, and removal efficiency for the target pollutants from the aqueous medium. The influence of the initial solution pH (pH_0_) on the adsorption efficiency of nitrates and phosphates onto SBAC_100_MgFe and SBAC_500_MgFe composites is shown in Figure 4a,b, respectively. At first glance, in Figure 4a,b, it could be inferred that changes in the removal efficiencies and adsorption capacities of phosphate follow similar patterns for both SBAC-MgFe composites. However, the phosphate removal of the SBAC_100_MgFe composite is more sensitive to pH_0_ changes when compared to the SBAC_500_MgFe composite. The phosphate removal efficiency of SBAC_100_MgFe varied between 79% (pH_0_ = 3) and 39% (pH_0_ = 12), with a standard deviation (SD) of ±13.6, while the SBAC_500_MgFe composite indicated phosphate removal levels in the range of 76% (pH_0_ = 3) to 67% (pH_0_ = 6), with a low SD of ±3.27. For the SBAC_100_MgFe composite, 21% and 23% reductions in phosphate removal efficiency were noticed when pH_0_ shifted from 3 to 4 and from 8 to 10, respectively. As in the case of phosphate removal, variation patterns in the removal efficiencies and adsorption capacities of nitrate for both SBAC-MgFe composites are almost similar. Yet again, it is observed that the nitrate removal of the SBAC_100_MgFe composite is more pH_0_-dependent in comparison with the SBAC_500_MgFe composite. The nitrate removal efficiency of SBAC_100_MgFe fluctuated between 36% (pH_0_ = 3) and 0.5% (pH_0_ = 12), with a higher SD of ±12.1. In addition, nitrate removal exhibited a continuous decrease when the pH_0_ increased. While both composites provided very similar removal efficiencies for phosphate removal, the SBAC_500_MgFe composite provided deficient nitrate removal performance (4% max at pH_0_ = 3) compared to the SBAC_100_MgFe composite (36% max at pH_0_ = 3). The equilibrium pH after phosphate and nitrate adsorption onto the SBAC-MgFe composites is displayed in Figure S2. The results show that the equilibrium pH was significantly increased to 6 and 7 at initial pH levels of 3 and 4, respectively. An increase in pH after adsorption was due to the anion exchange of OH-bonded metal groups (Mg-OH or Fe-OH) with phosphate and nitrate species in the solution. Similar behavior was reported in similar studies for the adsorption of phosphates and nitrates from carbon-modified LDH composites [14]. To summarize, SBAC_100_MgFe demonstrated the best removal performance and adsorption capacity for both anions at pH_0_ = 3, even though its uptake performance was highly dependent on pH_0_ changes.

Point of zero charge (pHpzc) values of SBAC-MgFe composites were predicted using the pH-drift method in the pH range 2–10, and the results are demonstrated in Figure 4c. The pHpzc values of the SBAC_100_MgFe and SBAC_500_MgFe composites were computed as 7.54 and 6.57, respectively. Generally, the surface charge of adsorbents becomes protonated when pH < pHpzc. It can be inferred from Figure 4 that the SBAC_100_MgFe composite provided better removal rates for phosphate and nitrate as the pH_0_ < pHpzc = 7.54. Under acidic conditions, the present forms of phosphate (HPO_4_^2−^ and H_2_PO_4_^−^) and nitrate (NO_3_^−^) in the solution electrostatically interact with the positively charged surface of SBAC_100_MgFe, resulting in better removal rates and adsorption capacities for both anions by the SBAC_100_MgFe composite [38,39]. The removal performance of phosphate and nitrate by the SBAC-MgFe composite is observed to be better at pH_0_ = 8 < pHpzc. At pH_0_ = 2–8, phosphate and nitrate species had lower adsorption free energy and could easily interact with the composite surface, forming metal–ligand complexes [38]. On the contrary, the SBAC_100_MgFe composite indicated a decreased removal efficiency when pH_0_ > 8. This is due to the electrostatic repulsion between anions and the deprotonated surface of the composite. At a high pH_0_ > 8.5, the solubility of anions, particularly phosphate, significantly reduced [40]. This might facilitate the chemical precipitation of phosphate on the SBAC-MgFe composite’s surface. Based on the results obtained herein, the optimum initial solution pH was selected as 3, and this was applied in the further adsorption experiments.

#### 3.2.2. Adsorbent Dosage

**Figure 5:**
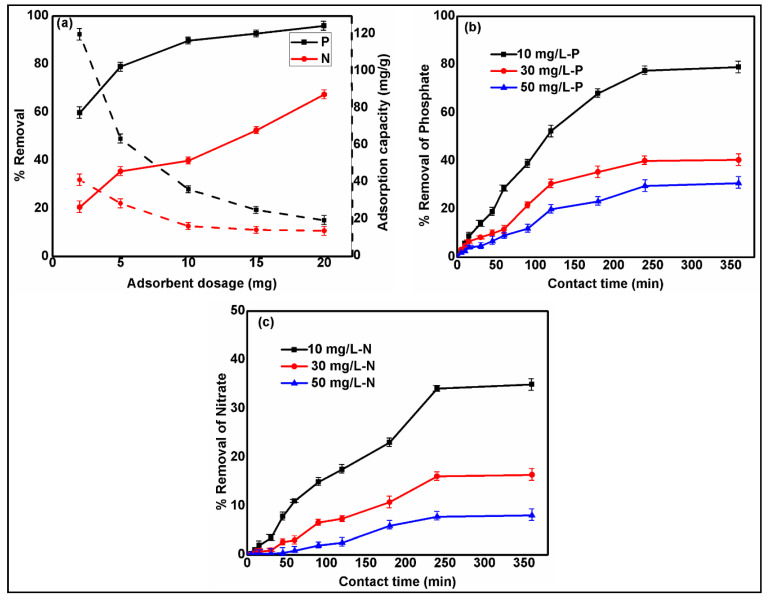
Effect of SBAC_100_MgFe composite dosage on nitrate and phosphate removals (**a**). Effect of contact time on the removal of phosphate (**b**) and nitrate (**c**) using SBAC_100_MgFe composite.

The adsorption dosage is of the utmost importance for the eco-effective treatment of pollutants from wastewater streams. The impact of adsorbent dosage on the adsorption efficiency for nitrate and phosphate was studied at varying dosages of SBAC_100_MgFe, between 2–20 mg at (i) pH = 3; (ii) initial anion concentration of 10 mg/L; (iii) working temperature of 298 K. The effects of composite dosage on removal rates and adsorption capacities for nitrate and phosphate are plotted in Figure 5a. The SBAC_100_MgFe composite demonstrated similar removal rate and adsorption capacity trends for both anions. The maximum adsorption capacities of phosphate and nitrate were found to be 120 mg/g and 41 mg/g, respectively, at the lowest composite dosage of 2 mg. The further increase in the dosage resulted in lower adsorption capacities for both ions. On the other hand, the phosphate removal rate increased from 60% to 90% when the dosage was increased from 2 mg to 10 mg, and then the phosphate removal rate did not show a significant improvement when the dosage reached 20 mg. Nitrate removal efficiency exhibited a linear increase, where it enhanced from 21% to 67% as a result of increasing the dosage from 2 mg to 20 mg. The highest increase in removal rate for phosphate and nitrate was found to be 20% and 15%, respectively, when the composite dosage was increased from 2 mg to 5 mg. Based on the removal efficiency and adsorption capacity results and economic considerations, the optimum adsorbent dosage was selected as 5 mg, which was used in the following adsorption tests.

#### 3.2.3. Contact Time and Kinetic Modeling

The effect of contact time on the removal efficiencies of phosphate and nitrate were investigated within the time interval of 0 to 360 min at three different levels of initial anion centration (10, 30, and 50 mg/L). At the same time, other parameters were kept constant: (i) pH = 3; (ii) stirring rate = 250 rpm; (iii) temperature = 298 K, composite dosage = 5 mg. The effects of contact time on the removal rates of phosphate and nitrate are shown in Figure 5b,c, respectively. The removal efficiencies of both ions demonstrated very similar tendencies for increasing contact time; they increased linearly until the contact time of 250 min, and then no further increases were noticed. It could be concluded from Figure 5b,c that the adsorption equilibrium time of both anions is 250 min. This adsorption equilibrium time could be ascribed to the fast interaction between anions and actively available binding units on the SBAC_100_MgFe composite.

The most well-known and practiced kinetic models, pseudo-first-order (PFO) and pseudo-second-order (PSO), were adopted when modeling the experimental adsorption kinetic dataset in order to assess the adsorption behavior of nitrate and phosphate ions on the SBAC_100_MgFe composite. The kinetic parameters and their corresponding correlation coefficients (*R*^2^) are given in Table 3. The kinetic modeling results revealed that the PFO model indicated better fitting with the obtained data (*R*^2^ = 0.930–0.994) compared to those of the PSO model (*R*^2^ = 0.115–0.901) within the temperature range of 298–308 K [23]. Besides this, the experimentally obtained *q_e_* values of nitrate and phosphate adsorption are very close to the *q_e_* values estimated using the PFO model. This behavior could be ascribed to the physical processes, electrostatic attraction, and ion exchange, mainly governing the nitrate and phosphate adsorption onto the SBAC_100_MgFe composite. Moreover, at 10 mg/L nitrate and phosphate concentrations, the adsorption rate of phosphate and nitrate was faster in comparison with previous studies. This might be due to lower PFO model rate constants (*k_1_*) 0.0184 and 0.0416 g/(mg min) of nitrate and phosphate ions, respectively. For instance, Yuan et al. [41] reported that *k_1_* = 0.10 min^−1^ and Wei et al. [42] stated that *k_1_* = 0.36 min^−1^ for phosphate adsorption, values which are higher than those obtained in the present study. Accordingly, the SBAC_100_MgFe composite has a fast adsorption rate at low initial nitrate and phosphate concentrations and can reach adsorption equilibrium faster.

To identify the main sorption stages of phosphate and nitrate, the kinetic dataset was further fitted to the film diffusion and intraparticle diffusion models [43]. The film diffusion model represents the overall effect of external diffusion of phosphate and nitrate ions from solution to SBAC_100_MgFe composite surface. As shown in Figure S3a,b, at all studied concentrations and times (10–60) min, the plot of −log(1 − *q_t_*/*q_e_*) against time is almost linear, which indicates that the film diffusion substantially contributes to the rate of adsorption of phosphate and nitrate ions [44]. Moreover, the sorption of phosphate and nitrate followed three main stages (Figure S3c,d): the first stage (I) indicates a fast adsorption rate associated with external diffusion and interaction with adsorbent surface functional groups; the second stage (II) likely represents the internal diffusion of phosphate and nitrate ions from the solution to the pores of the adsorbent; the third stage (III) is the equilibrium stage due to saturation of active binding sites. Interestingly, it was observed that stage I (film diffusion) and stage II (intraparticle diffusion) significantly contribute to the entire sorption kinetics of phosphate and nitrate ions on the SBAC_100_MgFe composite.

#### 3.2.4. Coexisting Ions

**Figure 6:**
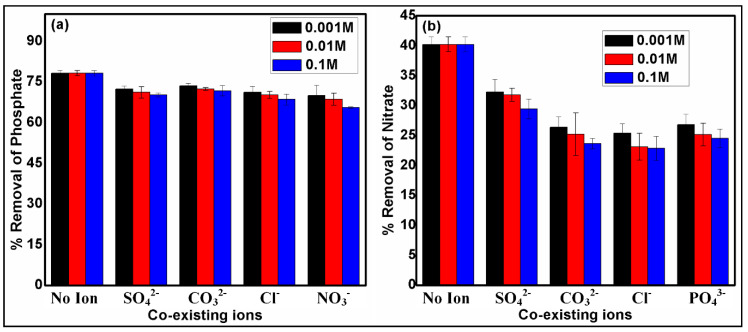
Effect of coexisting ions on the removal of phosphate (**a**) and nitrate (**b**) using SBAC_100_MgFe composite.

The presence of various ions in wastewater streams can affect the removal efficiencies of the targeted pollutants [45]. The influence of the coexisting anions (Cl^−^, NO_3_^−^, CO_3_^2−^, SO_4_^2−^, and PO_4_^3−^) with three concentration levels (0.001 M, 0.01 M, and 0.1 M) on the removal performance of nitrate and phosphate anions by SBAC_100_MgFe is depicted in Figure 6a,b. The percentage reductions in removal performances due to the coexisting ions were computed to be 7.9–17.4% (SD = 3.05%) for nitrate and 4.7–12.7% (SD = 2.06%) for phosphate. Removal efficiencies of nitrate and phosphate reduced as the concentration of coexisting anions increased. The removal efficiency of nitrate decreases because the individual coexisting ions at a 0.1 M concentration could be sorted in descending order: Cl^−^ (17.4%) > CO_3_^2−^ (16.6%) > PO_4_^3−^ (15.7) > SO_4_^2−^ (10.8%). The Cl^−^, CO_3_^2−^, SO_4_^2−^, and PO_4_^3−^ ions interact with the surface-active binding sites of the composite and compete with nitrate ions during the adsorption process, leading to a moderate reduction in the removal rate of nitrate ions. The effects of coexisting ions (0.1 M) resulted in percentage decreases for phosphate removal in the following order: NO_3_^−^ (12.7%) > Cl^−^ (9.7%) > SO_4_^2−^ (8.0%) > CO_3_^2−^ (6.5%). The competition between phosphate and studied coexisting ions through the adsorption process is remarkably lower in comparison with nitrate adsorption in the presence of interfering ions. It can be concluded from the effects of coexisting ions that the affinity of phosphate ions towards the adsorption sites of the SBAC_100_MgFe composite are stronger than those of nitrate ions. It is also important to mention that the removal efficiency of both nitrate and phosphate by the SBAC_100_MgFe composite considerably decreases in the presence of a high concentration of Cl^−^ ions. The obtained outcomes herein are quite similar to previous works that targeted nitrate and phosphate removal by AC-based LDHs in coexistence with Cl^−^, CO_3_^2−^, and SO_4_^2−^ ions [46,47].

### 3.3. Adsorption Isotherm Modeling Studies

**Figure 7:**
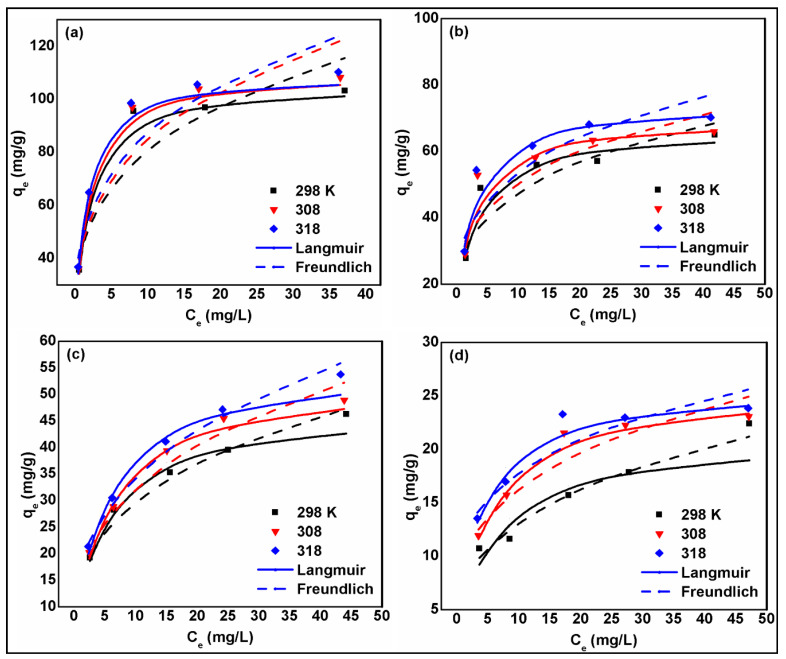
Plots of non-linear isotherm model results at different temperatures for phosphate adsorption at pH 3 (**a**) and at pH 6 (**b**) and for nitrate adsorption at pH 3 (**c**) and at pH 6 (**d**) onto SBAC_100_MgFe composite.

The experimental dataset obtained from the equilibrium studies at different operational temperatures (298 K, 308 K, and 318 K) and two levels of pH (3 and 6) were fitted to non-linear Langmuir [25] and Freundlich [26] models in order to elucidate the adsorptive interaction between nitrate/phosphate molecules on the SBAC_100_MgFe composite (Figure 7). The results of adsorption isotherm models provided in Table 4 revealed that the adsorption isotherm data of phosphate and nitrate ions were described better by the Langmuir model (0.974 < *R*^2^ < 0.989) and the Freundlich model (0.977 < *R*^2^ < 0.995), respectively, although both models generally performed well.

The separation factor (*R_L_*) was found in the ranges (0.20–0.02) and (0.45–0.05) for phosphate and nitrate adsorption at (298–318 K), respectively, indicating favorable adsorption (Figure S4). Interestingly, for both ions, the maximum monolayer adsorption capacity obtained from the Langmuir model increased with the rising temperature and reduced with increasing initial solution pH. Accordingly, the maximum uptake capacities for phosphate and nitrate ions onto the SBAC_100_MgFe that were achieved at the highest investigated temperature level of 318 K were (110 and 73.4) mg/g and (54.5 and 25.7) mg/g at pH 3 and pH 6, respectively. The higher sorption affinity at the low pH value is primarily associated with strong electrostatic attraction, leading to better complexation between phosphate and nitrate anions with protonated surface functionalities. Whereas at a pH value of 6, the surface of SBAC_100_MgFe had abundant hydroxyl ions, which might cause repulsive interactions with phosphate and nitrate anions, resulting in a lower adsorption capability. Moreover, the formation of Mg or Fe complex minerals with phosphate exhibits a comparatively much lower solubility than nitrate minerals, which resulted in higher sorption of phosphate than nitrate by the SBAC-MgFe composites [48].

The higher Langmuir constant (*K_L_*) values for the phosphate compared to those of the nitrate indicate a higher affinity of the phosphate ion, which suggests a stronger interaction with the SBAC_100_MgFe composite functional groups. This is manifested in the higher uptake capacities for the phosphate ions for all the operational conditions investigated. The Freundlich isotherm model’s intensity of adsorption and heterogeneity parameter (i.e., *1/n*) ranging from 0 to 1 indicates that both ions were favorably adsorbable onto the SBAC_100_MgFe composite. However, the lower values for phosphate further demonstrate superior favorable behavior compared with the nitrate. Inferably, the excellent adsorptive performance of the SBAC_100_MgFe indicates the successful intercalation of the SBAC_100_ with the MgFe/LDH to produce an effective composite for efficient nutrient removal from polluted water and wastewater.

### 3.4. Thermodynamic Modeling Studies

The effect of temperature on the adsorption of phosphate and nitrate onto the SBAC_100_MgFe composite was studied at three different temperature values of 298, 308, and 318 K. The adsorbed amounts of phosphate and nitrate ions increased by 2.90% (pH 3), 9.79% (pH 6), 7.61% (pH 3), and 31.6% (pH 6), respectively, when the solution temperature increased from 298 to 318 K, suggesting the endothermic nature of the adsorption process. The thermodynamic parameters, such as standard free Gibbs energy (Δ*G*), standard enthalpy change (Δ*H*), and standard entropy change (Δ*S*), were further computed in order to reveal the adsorption mechanism and feasibility of the adsorption by using the van’t Hoff equations (Equations (3) and (4)) [22,49,50].

(3) $$\ln\left(K_{d}\right)=-\frac{\Delta H}{R}\frac{1}{T}+\frac{\Delta S}{R}$$

(4) ΔG=ΔH−TΔS

where *K_d_* is the thermodynamic equilibrium constant calculated by plotting ln(*q_e_*/*C_e_*) against *q_e_* and extrapolating *q_e_* to zero; *R* is the universal gas constant (8.314 J/mol K); *T* is the absolute temperature (K). Δ*H* and Δ*S* are estimated from the slope and intercept in the plot of ln (*K_d_*) versus *1/T*.

All the thermodynamic parameters computed for phosphate and nitrate adsorption at pH 3 and pH 6 within the temperature range of 298–318 K are listed in Table 5. Negative Δ*G* values showed that the adsorption processes of phosphate and nitrate were spontaneous. Δ*G* values decreased from −8.43 to −9.35 kJ/mol (phosphate pH 3), −6.27 to −7.48 kJ/mol (phosphate pH 6), −3.64 to −4.21 kJ/mol (nitrate pH 3), and from −0.76 to −2.06 kJ/mol, indicating that increasing the solution temperature enhanced the spontaneity of the adsorption processes for both phosphate and nitrate ions. Positive Δ*H* values of phosphate and nitrate adsorption suggested the endothermic nature of the processes. Positive Δ*S* values implied that the movement of phosphate and nitrate ions from the aqueous phase to the surfaces of the SBAC_100_MgFe composite increased the randomness of the solid–liquid interface during the adsorption processes. In addition to this, the uptakes of phosphate and nitrate ions from the aqueous solution onto the composite were favorable processes. The required energy for the release of water molecules and NO_3_^2−^ ions from the interlayers of the composite exceeding the energy released due to the phosphate and nitrate ions attaching to the surface of the composite could explain the positive Δ*H* and Δ*S* values [51]. Higher Δ*H* and Δ*S* values were obtained for phosphate and nitrate adsorption at pH 6 than those found at pH 3. The assessment of Δ*S* values confirms that the SBAC_100_MgFe composite exhibits a higher affinity for both ions at pH 3 than pH 6 [52]. This could be associated with the different dominated interaction mechanisms of both ions with SBAC_100_MgFe, namely electrostatic attraction and ion exchange at pH 3 and 6, respectively.

### 3.5. Mechanism Insight

**Figure 8:**
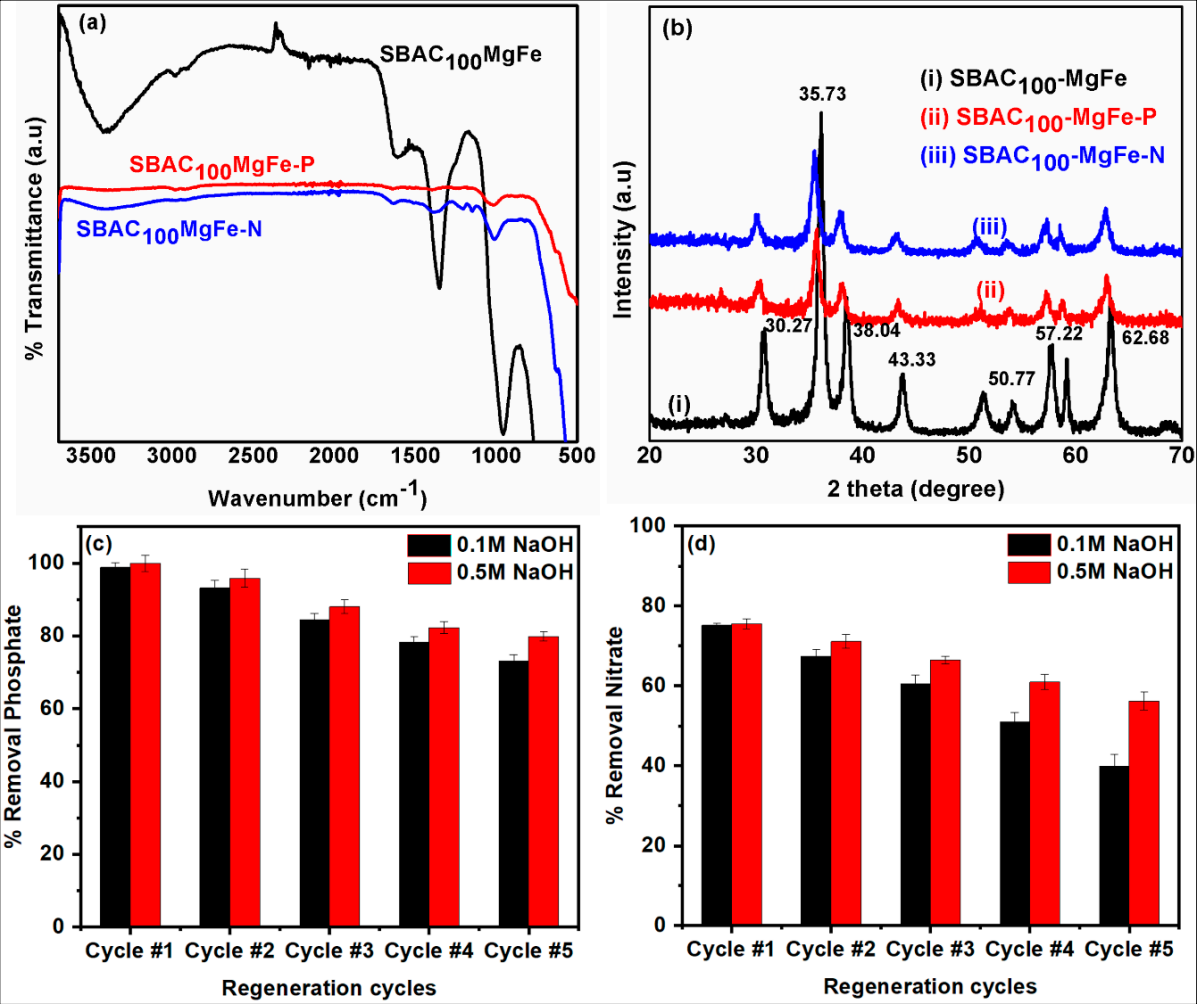
FT-IR (**a**) and XRD (**b**) images of SBAC_100_MgFe composite after phosphate and nitrate adsorption; reusability performance of SBAC_100_MgFe composite for phosphate (**c**) and nitrate (**d**) removal after regeneration via NaOH solutions.

Mechanistic studies are very significant in order to gain insights into the possible adsorption mechanisms between the adsorbent and adsorbate. For this reason, FT-IR and XRD analyses were carried out for the pristine and spent SBAC_100_MgFe composites (Figure 8a,b). Figure 8a depicts the FT-IR spectra of the SBAC_100_MgFe composite before and after adsorption of phosphate and nitrate. The absorption bands around 3400 cm^−1^ correspond to -OH stretching vibrations due to the structural -OH groups and interlayered H_2_O molecules. The absorption band that appeared at 1380 cm^−1^ could be attributed to the nitrate ions that intercalated in the interlayer space of the composite after nitrate adsorption [53,54,55]. A significant decrease in the intensity of NO_3_^−^ is attributed to the contribution of the anion exchange mechanism between interlayered NO_3_^−^ with phosphate and nitrate species. Similar behavior was reported by Hu et al. [38] after phosphate removal by CuAl/biochar. The formation of a broad peak at 1050 cm^−1^ could be assigned to P-O stretching vibrations, suggesting that -OH groups are replaced by the adsorbed phosphate and nitrate [22,51,56,57,58].

The comparison of the XRD spectra of pure and spent SBAC_100_MgFe composites are displayed in Figure 8b. The forms of SBAC_100_MgFe composite still indicated a similar crystallographic structure after being exposed to phosphate and nitrate ions. This might be attributed to the fact that the interaction between phosphate and nitrate was dominated by the inner sphere galleries of the composite, which resulted in the preservation of the crystallographic structure [55,59]. However, the peak intensities of the composite dramatically decreased, and the peak positions shifted slightly to the left after phosphate and nitrate adsorption, in comparison with the pre-adsorption composite [60]. These changes could be explained by the fact that electrostatic attractions between anion exchange sites on the SBAC_100_MgFe composite’s surface and phosphate/nitrate anions caused changes in both charge densities and interlayer spaces of the composite [60,61].

### 3.6. Regeneration Studies and Reusability Performance

The reusability of an adsorbent is a critical factor in designing an eco-friendly and sustainable adsorption process for the treatment of pollutants from wastewaters with high influent flow rates. In this context, sequential cycles of adsorption/desorption experiments were conducted to determine the reusability performance of the SBAC_100_MgFe composite. NaOH solutions with 0.1 and 0.5 M concentrations were used to regenerate the spent composite to be tested for the subsequent adsorption/desorption sequence. The magnitude of nitrate and phosphate anions removed after every adsorption/desorption cycle (*n*) is called the removal efficiency (*RE (%)*) and is computed using Equation (5) [62]:

(5) $$RE\;\left(\%\right)=\frac{\%\;Removal\;of\;nitrate\;or\;phosphate\;ions\;\left(mg\right)\;at\;run\;\left(n+1\right)}{\;\%\;Removal\;nitrate\;or\;phosphate\;ions\;\left(mg\right)\;at\;run\;\left(n\right)}\times 100$$

Figure 8c,d depict the removal efficiencies of nitrate and phosphate ions for each regeneration cycle, respectively. As shown in Figure 8c,d, the SBAC_100_MgFe composite indicated a gradual decrease in removal efficiency for both anions after each regeneration cycle. The reductions in phosphate removal were found on average to be 6.5% (±1.4) and 5.0% (±2.0) after each regeneration cycle using 0.1 and 0.5 M NaOH, respectively. For nitrate removal, the average decreases in efficiency were 8.8% (±1.6) and 4.8% (±0.4) after each cycle using 0.1 and 0.5 M NaOH, respectively. It could be inferred that using 0.5 M NaOH in the regeneration process for the SBAC_100_MgFe composite provided better reusability performance for both anions than that of using 0.1 M NaOH. At the end of the fifth sequential adsorption/desorption cycle, the phosphate and nitrate removal efficiencies were reduced from 99.9% to 79.9% and from 75.5% to 56.2%, respectively, when using 0.5 M NaOH for regeneration. The results obtained for regeneration studies applied for the SBAC_100_MgFe composite revealed that active binding sites on the SBAC_100_MgFe composite could be easily and effectively restored, especially for phosphate adsorption, and the newly fabricated SBAC_100_MgFe composite in this work has an exceptional reusability performance for phosphate and nitrate treatment in wastewater streams.

### 3.7. Removal Performance of SBAC-MgFe Composite in Real Wastewater

The phosphate and nitrate removal performance of the SBAC_100_MgFe composite was tested on a real wastewater sample which was obtained from the domestic wastewater treatment plant located in the main campus area of Imam Abdurrahman Bin Faisal University (26°23’47.64” N, 50°12’8.93” E). The composition of wastewater is summarized in Table S1. The phosphate and nitrate compositions of the influent wastewater treatment plant samples were quantified as 4.02 mg/L and 0.595 mg/L, respectively. The following operational conditions were used to test the removal performance of the SBAC-MgFe composite on the wastewater sample: (i) composite dosage, 5 mg; (ii) working volume, 40 mL; (iii) contact time, 250 min; (iv) pH_0_ = 3; (v) temperature = 298 K, stirring rate = 250 rpm. Adsorption experiments were applied in triplicate shortly after the samples were collected. Phosphate and nitrate removal efficiencies were found to be 78.7% (±0.03) and 37.8% (±0.72), respectively. The composite indicated phosphate and nitrate adsorption capacities of 22.9 mg/g (±2.15) and 0.993 mg/g (±0.57), respectively. The results suggest that the fabricated SBAC_100_Mg-Fe composite is a promising adsorbent material for the effective treatment of phosphate and nitrate in real wastewaters.

### 3.8. Comparison with Other Carbon-Based LDH Composites and Cost Estimation

A comparison of adsorption capacities of previously reported carbon-based LDH composites with the magnetic SBAC_100_MgFe composite synthesized in this work for the treatment of phosphate and nitrate ions is shown in Table 6. As illustrated, the sorption capacity of the SBAC_100_MgFe composite is relatively higher or similar to previously reported studies of LDH/carbon materials. This suggests that the intercalation of sewage sludge-activated carbon within layers of MgFe LDH via the coprecipitation method resulted in an eco-friendly and sustainable material exhibiting suitable characteristics for the better uptake of phosphate and nitrate anions from wastewater streams. Thus, the magnetic SBAC_100_MgFe LDH composite could be a potential adsorbent material for phosphate and nitrate treatment from wastewaters.

One of the most significant factors in wastewater treatment is the cost estimation of the treatment method, considering its economic feasibility and sustainability. The wastewater treatment cost of various adsorption methods usually ranges between 5 and 200 US$/m^3^ of the treated water [68]. In addition to this, the cost of activated carbon (AC)-based adsorbent materials existing in nature was reported to be between 0.02 and 20 US$ per kg [69,70]. Commercial ACs cost within the range of 2 and 20 US$ per kg [69,71]. The cost of the magnetic SBAC_100_MgFe composite produced in this study was estimated to be 45 US$ per kg, which is higher than those of commercially and naturally existing AC-based adsorbent materials. However, phosphate and nitrate treatment cost using the magnetic SBAC_100_MgFe composite, with remarkably outstanding adsorption capacities for phosphate and nitrate (110 mg/g and 55 mg/g, respectively), was estimated to be 2 US$/m^3^ for wastewater effluent advanced treatment. This treatment cost is significantly lower than those of previously reported adsorption methods due to the superior adsorption capacity and outstanding reusability performance of the magnetic SBAC_100_MgFe composite.

## 4. Conclusions

This study focused on the design, fabrication, and application of a unique, magnetically separable, and eco-friendly adsorbent material derived from waste sludge for the effective treatment of phosphate and nitrate pollutants from wastewaters. Waste sludge-based activated carbons were intercalated with Mg-Fe/LDH using the coprecipitation method. The FT-IR, XRD, SEM, TEM, BET, and VSM characterization results revealed that the SBAC_100_MgFe composite indicated (i) significant improvement in functional groups; (ii) homogeneous and efficient dispersion of MgFe/LDH within the AC matrix; (iii) a highly mesoporous structure; (iv) paramagnetic characteristics, providing fast and easy removal. The studies investigating the effects of initial pH, adsorbent dose, and contact time specified that the SBAC_100_MgFe composite provided the best removal performance for both phosphate and nitrate pollutants at an initial pH of 3.0, 5.0 mg of composite mass, and 250 min of contact time. The maximum percentage reductions in removal performances were found to be 17.4% for nitrate and 12.7% for phosphate in the presence of coexisting ions with a 0.1 M concentration. The kinetic modeling results revealed that the pseudo-first-order model indicated better fitting with the experimental adsorption datasets of both anions, suggesting that the physical processes, such as electrostatic attraction and ion exchange, dominate the adsorption of nitrate and phosphate onto the SBAC_100_MgFe composite. The adsorption isotherm data of phosphate and nitrate ions were better described by the Langmuir and the Freundlich models, respectively, where the maximum adsorption capacity for phosphate is 110 mg/g, while a maximum adsorption capacity of 54.5 mg/g is computed for nitrate. The thermodynamic modeling results showed that the adsorption process of phosphate and nitrate is spontaneous, endothermic, and favorable. The post-adsorption characterization results demonstrated that the adsorbate–adsorbent surface charge interactions suggest the involvement of hydrogen bonding and π-π interactions, predominantly via the physisorption process (Δ*G*° = −0.76 to −4.21 kJ/mol for nitrate and Δ*G*° = −6.27 to−9.35 kJ/mol for phosphate). The outstanding regeneration performance of the SBAC_100_MgFe composite was achieved even after five sequences of adsorption/desorption cycles, suggesting the potential of its easy reusability and recyclability. The tests on real wastewater effluent samples demonstrated that the fabricated composite can be used as a promising advanced treatment adsorbent for the effective removal of phosphate and nitrate pollutants from domestic or industrial wastewaters effluents.

## Figures and Tables

**Table 1:** Mix design mass and percent composition of SBAC and MgFe in SBAC-MgFe composites.

Composite	SBAC	Mg(II) Salt	Fe (III) Salt	SBAC (Carbon)	Mg(II)	Fe(III)
	Theoretical (g)			Actual (%)		
SBAC_100_MgFe	0.1	2.54	4.04	12.6	13.3	55.3
SBAC_500_MgFe	0.5	2.54	4.04	20.8	8.2	39.6

**Table 2:** Textural properties of SBAC-MgFe composites.

Textural Properties	SBAC_100_MgFe	SBAC_500_MgFe
BET surface area (m^2^/g)	169	257
Pore volume (cm^3^/g)	0.21	0.23
Pore radius (based on BJH) (nm)	1.82	1.72

**Table 3:** Parameters of kinetic models for phosphate and nitrate removal by SBAC_100_MgFe composite.

Pollutant	*C*_0_		Pseudo-First-Order			Pseudo-Second-Order		
			$ln\left(q_{e}-q_{t}\right)=lnq_{e}-k_{1}t$			$\frac{t}{q_{t}}=\frac{t}{q_{e}}+\frac{1}{k_{2}q_{e}^{2}}$		
		*q_e_ (exp)*	*q_e_*	*k*_1_	*R* ^2^	*q_e_*	*k*_2_ × 10^−5^	*R* ^2^
Phosphate	10	63.1	67.7	0.0416	0.992	99.0	6.03	0.899
	30	95.9	99.7	0.0451	0.990	135	6.07	0.901
	50	113	115	0.0161	0.994	170	3.20	0.703
Nitrate	10	27.9	31.9	0.0184	0.972	15.0	17.5	0.219
	30	39.3	42.5	0.0140	0.978	20.2	13.1	0.115
	50	32.2	43.1	0.0322	0.930	12.8	21.1	0.303

**Table 4:** Parameters of non-linear Langmuir and Freundlich isotherm models for phosphate and nitrate removal by SBAC_100_MgFe composite at pH 3 and pH 6.

Pollutant	T (K)	Langmuir $q_{e}=\frac{q_{max}bC_{e}}{1+bC_{e}}$			Freundlich $q_{e}=K_{F}C_{e}^{1/n}$		
		*q_max_*	*K_L_*	*R* ^2^	*K_F_*	*1/n*	*R* ^2^
Phosphate (pH 3)	298	104.1	0.89	0.989	46.1	0.25	0.927
	308	108.5	0.91	0.985	47.7	0.26	0.935
	318	109.9	1.12	0.974	50.3	0.25	0.944
Phosphate (pH 6)	298	65.5	0.51	0.981	28.1	0.23	0.932
	308	68.8	0.57	0.977	30.5	0.23	0.915
	318	73.4	0.58	0.981	32.0	0.23	0.917
Nitrate (pH 3)	298	46.3	0.25	0.988	15.1	0.30	0.993
	308	51.7	0.24	0.987	15.8	0.31	0.988
	318	54.5	0.23	0.981	16.7	0.32	0.995
Nitrate (pH 6)	298	20.8	0.21	0.925	6.66	0.29	0.981
	308	25.4	0.23	0.999	8.94	0.26	0.977
	318	25.7	0.31	0.987	10.7	0.22	0.977

**Table 5:** Thermodynamic parameters for phosphate and nitrate removal by SBAC_100_MgFe composite at pH 3 and pH 6.

	*T (K)*	*K_d_*	Δ*G* (kJ/mol)	Δ*H* (kJ/mol)	Δ*S* (J/mol K)
Phosphate (pH 3)	298	30.0	−8.43		
	308	31.8	−8.86	5.30	46.0
	318	34.3	−9.35		
Phosphate (pH 6)	298	12.6	−6.27		
	308	15.5	−7.01	11.8	60.7
	318	16.9	−7.48		
Nitrate (pH 3)	298	4.35	−3.64		
	308	4.50	−3.85	4.90	28.5
	318	4.92	−4.21		
Nitrate (pH 6)	298	1.36	−0.76		
	308	1.95	−1.71	18.8	65.8
	318	2.18	−2.06		

**Table 6:** Phosphate and nitrate adsorption capacities of various carbon-based layered double hydroxide (LDH) composites.

Adsorbent	Adsorbate	pH	Adsorption Capacity (mg/g)	Reference
Sugar cane leaves biochar/(4:1) MgAl	Phosphorous	3	81.8	[63]
Pinecone flakes biochar/MgFe	Phosphorous	2	17.5	[56]
Tobacco stalk biochar/MgAl	Phosphate	2	41.2	[64]
Rice husk biochar/MgAl calcined	Phosphate	4	121	[65]
Cabbage biochar/MgAl calcined	Phosphate	2	127	[66]
Rape biochar/MgAl calcined	Phosphate	2	133	[66]
Date palm fronds biochar/MgAl	Phosphate/Nitrate	3	146/31.9	[67]
Sewage sludge-activated carbon/MgFe LDH	Phosphate/Nitrate	3	104/46.3	This study

## Supplementary Materials

The following are available online at https://www.mdpi.com/2079-4991/10/7/1361/s1: Figure S1: Electron images and elemental composition spectrums of composites by SEM-EDX technique: (**a**) SBAC_100_MgFe, (**b**) SBAC_500_MgFe, Figure S2: Equilibrium pH value of phosphate (**a**) and nitrate (**b**) after adsorption by SBAC-MgFe composites, Figure S3: Film diffusion (**a,b**) and intraparticle diffusion (**c,d**) model plots for adsorption of phosphate and nitrate on SBAC_100_MgFe composite, Figure S4: Plot of separation factor (R_L_) versus initial concentration at three different temperature values for phosphate and nitrate adsorption on SBAC_100_MgFe, Table S1: Composition of wastewater obtained from domestic wastewater treatment plant of Imam Abdulrahman Bin Faisal University.

## References

[1] Bhateria R., Jain D. (2016). Water quality assessment of lake water: A review. Sustain. Water Resour. Manag..

[2] Keeney D., Olson R.A. (1986). Sources of nitrate to ground water. Crit. Rev. Environ. Contr..

[3] Mainstone C.P., Parr W. (2002). Phosphorus in rivers—Ecology and management. Sci. Total Environ..

[4] Imhoff P.T., Nakhli S.A.A. (2017). Reducing Stormwater Runoff and Pollutant Loading with Biochar Addition to Highway Greenways.

[5] Chen L.F., Liang H.W., Lu Y., Cui C.H., Yu S.H. (2011). Synthesis of an attapulgite clay@carbon nanocomposite adsorbent by a hydrothermal carbonization process and their application in the removal of toxic metal ions from water. Langmuir.

[6] Khan S., Chao C., Waqas M., Arp H.P., Zhu Y.G. (2013). Sewage sludge biochar influence upon rice (*Oryza sativa* L) yield, metal bioaccumulation and greenhouse gas emissions from acidic paddy soil. Environ. Sci. Technol..

[7] Puga A., Abreu C., Melo L., Beesley L. (2015). Biochar application to a contaminated soil reduces the availability and plant uptake of zinc, lead and cadmium. J. Environ. Manag..

[8] Zubair M., Mu’azu N.D., Jarrah N., Blaisi N.I., Aziz H.A., Al-Harthi M.A. (2020). Adsorption Behavior and Mechanism of Methylene Blue, Crystal Violet, Eriochrome Black T, and Methyl Orange Dyes onto Biochar-Derived Date Palm Fronds Waste Produced at Different Pyrolysis Conditions. Water Air Soil Pollut..

[9] Shaheen S.M., Niazi N.K., Hassan N.E.E., Bibi I., Wang H., Tsang D.C.W., Ok Y.S., Bolan N., Rinklebe J. (2018). Wood-based biochar for the removal of potentially toxic elements in water and wastewater: A critical review. Int. Mater. Rev..

[10] Hibino T., Tsunashima A. (1998). Characterization of Repeatedly Reconstructed Mg−Al Hydrotalcite-like Compounds:  Gradual Segregation of Aluminum from the Structure. Chem. Mater..

[11] Gupta N.K., Saifuddin M., Kim S., Kim K.S. (2020). Microscopic, spectroscopic, and experimental approach towards understanding the phosphate adsorption onto Zn–Fe layered double hydroxide. J. Mol. Liq..

[12] Santos L.C., da Silva A.F., dos Santos Lins P.V., da Silva Duarte J.L., Ide A.H., Meili L. (2020). Mg-Fe layered double hydroxide with chloride intercalated: Synthesis, characterization and application for efficient nitrate removal. Environ. Sci. Pollut. Res..

[13] Liu C., Zhang M., Pan G., Lundehøj L., Nielsen U.G., Shi Y., Hansen H.C.B. (2019). Phosphate capture by ultrathin MgAl layered double hydroxide nanoparticles. Appl. Clay Sci..

[14] Tran H.N., Nguyen H.C., Woo S.H., Nguyen T.V., Vigneswaran S., Hosseini-Bandegharaei A., Rinklebe J., Kumar Sarmah A., Ivanets A., Dotto G.L. (2019). Removal of various contaminants from water by renewable lignocellulose-derived biosorbents: A comprehensive and critical review. Crit. Rev. Environ. Sci. Technol..

[15] Ivanets A., Kitikova N., Shashkova I., Matrunchik Y., Kul’bitskaya L., Sillanpää M. (2016). Non-acidic synthesis of phosphatized dolomite and its sorption behaviour towards Pb^2+^, Zn^2+^, Cu^2+^, Cd^2+^, Ni^2+^, Sr^2+^ and Co^2+^ ions in multicomponent aqueous solution. Environ. Technol. Innov..

[16] Chen H., Liu S., Liu T., Yuan Z., Guo J. (2020). Efficient nitrate removal from synthetic groundwater via in situ utilization of short-chain fatty acids from methane bioconversion. Chem. Eng. J..

[17] Ivanets A.I., Shashkova I.L., Kitikova N.V., Kul’bitskaya L.V., Matrunchik Y.V. (2015). Study of the interaction of mono-, di-, and trisubstituted sodium orthophosphates with thermally activated dolomite. Russ. J. Appl. Chem..

[18] Zubair M., Manzar M.S., Mu’azu N.D., Anil I., Blaisi N.I., Al-Harthi M.A. (2020). Functionalized MgAl-layered hydroxide intercalated date-palm biochar for Enhanced Uptake of Cationic dye: Kinetics, isotherm and thermodynamic studies. Appl. Clay Sci..

[19] Xue L., Gao B., Wan Y., Fang J., Wang S., Li Y., Muñoz-Carpena R., Yang L. (2016). High efficiency and selectivity of MgFe-LDH modified wheat-straw biochar in the removal of nitrate from aqueous solutions. J. Taiwan Inst. Chem. Eng..

[20] Yin Q., Wang R., Zhao Z. (2018). Application of Mg–Al-modified biochar for simultaneous removal of ammonium, nitrate, and phosphate from eutrophic water. J. Clean. Prod..

[21] You H., Li W., Zhang Y., Meng Z., Shang Z., Feng X., Ma Y., Lu J., Li M., Niu X. (2019). Enhanced removal of NO_3_-N from water using Fe-Al modified biochar: Behavior and mechanism. Water Sci. Technol..

[22] Mu’azu N.D., Zubair M., Jarrah N., Alagha O., Al-Harthi M.A., Essa M.H. (2020). Sewage Sludge ZnCl2-Activated Carbon Intercalated MgFe-LDH Nanocomposites: Insight of the Sorption Mechanism of Improved Removal of Phenol from Water. Int. J. Mol. Sci..

[23] Liadi M.A., Mu’azu N.D., Jarrah N., Zubair M., Alagha O., Al-Harthi M.A., Essa M.H. (2020). Comparative performance study of ZnCl_2_ and NaOH sludge based activated carbon for simultaneous aqueous uptake of phenolic compounds. Int. J. Environ. Anal. Chem..

[24] Ho Y.S., McKay G. (1998). A Comparison of Chemisorption Kinetic Models Applied to Pollutant Removal on Various Sorbents. Process Saf. Environ..

[25] Langmuir I. (1916). The constitution and fundamental properties of solids and liquids Part I Solids. J. Am. Chem. Soc..

[26] Freundlich H.M.F. (1906). Over the adsorption in solution. J. Phys. Chem..

[27] Peng Y., Sun Y., Sun R., Zhou Y., Tsang D.C.W., Chen Q. (2019). Optimizing the synthesis of Fe/Al (Hydr)oxides-Biochars to maximize phosphate removal via response surface model. J. Clean. Prod..

[28] Chen S., Huang Y., Han X., Wu Z., Lai C., Wang J., Deng Q., Zeng Z., Deng S. (2018). Simultaneous and efficient removal of Cr(VI) and methyl orange on LDHs decorated porous carbons. Chem. Eng. J..

[29] Mu’azu N.D., Jarrah N., Zubair M., Alagha O. (2017). Removal of phenolic compounds from water using sewage sludge-based activated carbon adsorption: A review. Int. J. Environ. Res. Public Health.

[30] Zubair M., Daud M., McKay G., Shehzad F., Al-Harthi M.A. (2017). Recent progress in layered double hydroxides (LDH)-containing hybrids as adsorbents for water remediation. Appl. Clay Sci..

[31] Kazeem T.S., Zubair M., Daud M., Mu’azu N.D., Al-Harthi M.A. (2019). Graphene/ternary layered double hydroxide composites: Efficient removal of anionic dye from aqueous phase. Korean J. Chem. Eng..

[32] Durrani S.K., Naz S., Mehmood M., Nadeem M., Siddique M. (2017). Structural, impedance and Mössbauer studies of magnesium ferrite synthesized via sol–gel auto-combustion process. J. Saudi Chem. Soc..

[33] Ivanets A.I., Srivastava V., Roshchina M.Y., Sillanpää M., Prozorovich V.G., Pankov V.V. (2018). Magnesium ferrite nanoparticles as a magnetic sorbent for the removal of Mn2+, Co2+, Ni2+ and Cu2+ from aqueous solution. Ceram. Int..

[34] Valeikiene L., Roshchina M., Grigoraviciute-Puroniene I., Prozorovich V., Zarkov A., Ivanets A., Kareiva A. (2020). On the Reconstruction Peculiarities of Sol–Gel Derived Mg2−xMx/Al1 (M = Ca, Sr, Ba) Layered Double Hydroxides. Crystals.

[35] Kang D., Yu X., Tong S., Ge M., Zuo J., Cao C., Song W. (2013). Performance and mechanism of Mg/Fe layered double hydroxides for fluoride and arsenate removal from aqueous solution. Chem. Eng. J..

[36] Morrell D.G. (2019). Catalysis of Organic Reactions.

[37] Xu Q., Wei Y., Liu Y., Ji X., Yang L., Gu M. (2009). Preparation of Mg/Fe spinel ferrite nanoparticles from Mg/Fe-LDH microcrystallites under mild conditions. Solid State Sci..

[38] Hu F.P., Wang M., Peng X.M., Qiu F.X., Zhang T., Dai H.L., Liu Z.M., Cao Z. (2018). High-efficient adsorption of phosphates from water by hierarchical CuAl/biomass carbon fiber layered double hydroxide. Colloids Surf. A Physicochem. Eng. Asp..

[39] Azam H.M., Alam S.T., Hasan M., Yameogo D.D.S., Kannan A.D., Rahman A., Kwon M.J. (2019). Phosphorous in the environment: Characteristics with distribution and effects, removal mechanisms, treatment technologies, and factors affecting recovery as minerals in natural and engineered systems. Environ. Sci. Pollut. Res. Int..

[40] Diaz O.A., Reddy K.R., Moore P.A. (1994). Solubility of inorganic phosphorus in stream water as influenced by pH and calcium concentration. Water Res..

[41] Yuan L., Qiu Z., Yuan L., Tariq M., Lu Y., Yang J., Li Z., Lyu S. (2019). Adsorption and mechanistic study for phosphate removal by magnetic Fe3O4-doped spent FCC catalysts adsorbent. Chemosphere.

[42] Wei A., Ma J., Chen J., Zhang Y., Song J., Yu X. (2018). Enhanced nitrate removal and high selectivity towards dinitrogen for groundwater remediation using biochar-supported nano zero-valent iron. Chem. Eng. J..

[43] Tan K.L., Hameed B.H. (2017). Insight into the adsorption kinetics models for the removal of contaminants from aqueous solutions. J. Taiwan Inst. Chem. Eng..

[44] Ivanets A.I., Shashkova I.L., Kitikova N.V., Morozov Y. (2016). The kinetic studies of the cobalt ion removal from aqueous solutions by dolomite-based sorbent. Int. J. Environ. Sci. Technol..

[45] Wei J., Meng X., Wen X., Song Y. (2020). Adsorption and recovery of phosphate from water by amine fiber, effects of co-existing ions and column filtration. J. Environ. Sci..

[46] Karthikeyan P., Elanchezhiyan S.S.D., Preethi J., Meenakshi S., Park C.M. (2020). Mechanistic performance of polyaniline-substituted hexagonal boron nitride composite as a highly efficient adsorbent for the removal of phosphate, nitrate, and hexavalent chromium ions from an aqueous environment. Appl. Surf. Sci..

[47] Berkessa Y.W., Mereta S.T., Feyisa F.F. (2019). Simultaneous removal of nitrate and phosphate from wastewater using solid waste from factory. Appl. Water Sci..

[48] Nzihou A., Sharrock P. (2010). Role of Phosphate in the Remediation and Reuse of Heavy Metal Polluted Wastes and Sites. Waste Biomass Valoriz..

[49] Anil I., Gunday S.T., Bozkurt A., Alagha O. (2020). Design of Crosslinked Hydrogels Comprising Poly(Vinylphosphonic Acid) and Bis[2-(Methacryloyloxy)Ethyl] Phosphate as an Efficient Adsorbent for Wastewater Dye Removal. Nanomaterials.

[50] Halajnia A., Oustan S., Najafi N., Khataee A.R., Lakzian A. (2012). The adsorption characteristics of nitrate on Mg–Fe and Mg–Al layered double hydroxides in a simulated soil solution. Appl. Clay Sci..

[51] Yan L.G., Yang K., Shan R.R., Yan T., Wei J., Yu S.J., Yu H.Q., Du B. (2015). Kinetic, isotherm and thermodynamic investigations of phosphate adsorption onto core-shell Fe(3)O(4)@LDHs composites with easy magnetic separation assistance. J. Colloid Interface Sci..

[52] Ivanets A.I., Srivastava V., Kitikova N.V., Shashkova I.L., Sillanpää M. (2017). Kinetic and thermodynamic studies of the Co(II) and Ni(II) ions removal from aqueous solutions by Ca-Mg phosphates. Chemosphere.

[53] Halajnia A., Oustan S., Najafi N., Khataee A.R., Lakzian A. (2013). Adsorption–desorption characteristics of nitrate, phosphate and sulfate on Mg–Al layered double hydroxide. Appl. Clay Sci..

[54] Tong X., Yang Z., Xu P., Li Y., Niu X. (2017). Nitrate adsorption from aqueous solutions by calcined ternary Mg-Al-Fe hydrotalcite. Water Sci. Technol..

[55] Yang Z., Zhang L., Xu P., Zhang X., Niu X., Zhou S. (2014). The adsorption of nitrate from aqueous solution onto calcined Mg/Fe hydrotalcite. Desalin. Water Treat..

[56] Bolbol H., Fekri M., Hejazi-Mehrizi M. (2019). Layered double hydroxide–loaded biochar as a sorbent for the removal of aquatic phosphorus: Behavior and mechanism insights. Arab. J. Geosci..

[57] Chitrakar R., Tezuka S., Hosokawa J., Makita Y., Sonoda A., Ooi K., Hirotsu T. (2010). Uptake properties of phosphate on a novel Zr-modified MgFe-LDH(CO(3)). J. Colloid Interface Sci..

[58] Kim T.-H., Lundehøj L., Nielsen U.G. (2020). An investigation of the phosphate removal mechanism by MgFe layered double hydroxides. Appl. Clay Sci..

[59] Sun X., Imai T., Sekine M., Higuchi T., Yamamoto K., Kanno A., Nakazono S. (2014). Adsorption of phosphate using calcined Mg_3_–Fe layered double hydroxides in a fixed-bed column study. J. Ind. Eng. Chem..

[60] Wan S., Wang S., Li Y., Gao B. (2017). Functionalizing biochar with Mg–Al and Mg–Fe layered double hydroxides for removal of phosphate from aqueous solutions. J. Ind. Eng. Chem..

[61] Sasai R., Norimatsu W., Matsumoto Y. (2012). Nitrate-ion-selective exchange ability of layered double hydroxide consisting of MgII and FeIII. J. Hazard. Mater..

[62] Baldermann A., Fleischhacker Y., Schmidthaler S., Wester K., Nachtnebel M., Eichinger S. (2020). Removal of Barium from Solution by Natural and Iron(III) Oxide-Modified Allophane, Beidellite and Zeolite Adsorbents. Materials.

[63] Li R., Wang J.J., Zhou B., Awasthi M.K., Ali A., Zhang Z., Gaston L.A., Lahori A.H., Mahar A. (2016). Enhancing phosphate adsorption by Mg/Al layered double hydroxide functionalized biochar with different Mg/Al ratios. Sci. Total Environ..

[64] He H., Zhang N., Chen N., Lei Z., Shimizu K., Zhang Z. (2019). Efficient phosphate removal from wastewater by MgAl-LDHs modified hydrochar derived from tobacco stalk. Bioresour. Technol. Rep..

[65] Lee S.Y., Choi J.-W., Song K.G., Choi K., Lee Y.J., Jung K.-W. (2019). Adsorption and mechanistic study for phosphate removal by rice husk-derived biochar functionalized with Mg/Al-calcined layered double hydroxides via co-pyrolysis. Compos. Part B Eng..

[66] Zhang Z., Yan L., Yu H., Yan T., Li X. (2019). Adsorption of phosphate from aqueous solution by vegetable biochar/layered double oxides: Fast removal and mechanistic studies. Bioresour. Technol..

[67] Alagha O., Manzar M.S., Zubair M., Anil I., Mu’azu N.D., Qureshi A. (2020). Comparative Adsorptive Removal of Phosphate and Nitrate from Wastewater Using Biochar-MgAl LDH Nanocomposites: Coexisting Anions Effect and Mechanistic Studies. Nanomaterials.

[68] Ali I., Asim M., Khan T.A. (2012). Low cost adsorbents for the removal of organic pollutants from wastewater. J. Environ. Manag..

[69] Gupta V.K. (2009). Application of low-cost adsorbents for dye removal—A review. J. Environ. Manag..

[70] De Gisi S., Lofrano G., Grassi M., Notarnicola M. (2016). Characteristics and adsorption capacities of low-cost sorbents for wastewater treatment: A review. Sustain. Mater. Technol..

[71] Rafatullah M., Sulaiman O., Hashim R., Ahmad A. (2010). Adsorption of methylene blue on low-cost adsorbents: A review. J. Hazard. Mater..

