# Supplementary material for: Magnetic Mg-Fe/LDH Intercalated Activated Carbon Composites for Nitrate and Phosphate Removal from Wastewater: Insight into Behavior and Mechanisms
Source: Nanomaterials (Basel). 2020 Jul 12;10(7):1361. doi: 10.3390/nano10071361 (PMC7407415; doi:10.3390/nano10071361)
Supplement: Supplementary file 1 [file nanomaterials-10-01361-s001.pdf]

nanomaterials

Article

Magnetic Mg-Fe/LDH Intercalated Activated Carbon Composites for Nitrate and Phosphate Removal from Wastewater: Insight into Behavior and Mechanisms

Omar Alagha *, Mohammad Saood Manzar, Mukarram Zubair, Ismail Anil, Nuhu Dalhat Mu'azu and Aleem Qureshi

Environmental Engineering Department, College of Engineering A13, Imam Abdulrahman Bin Faisal University, Main Campus, P.O. Box 1982, Dammam 34212, Saudi Arabia; msmanzar@iau.edu.sa (M.S.M.); mzzubair@iau.edu.sa (M.Z.); ianil@iau.edu.sa (I.A.); nmdalhat@iau.edu.sa (N.D.M.); aqureshi@iau.edu.sa (A.Q.)

* Correspondence: oaga@iau.edu.sa; Tel.: +966-50-661-65-32

Supplementary Materials

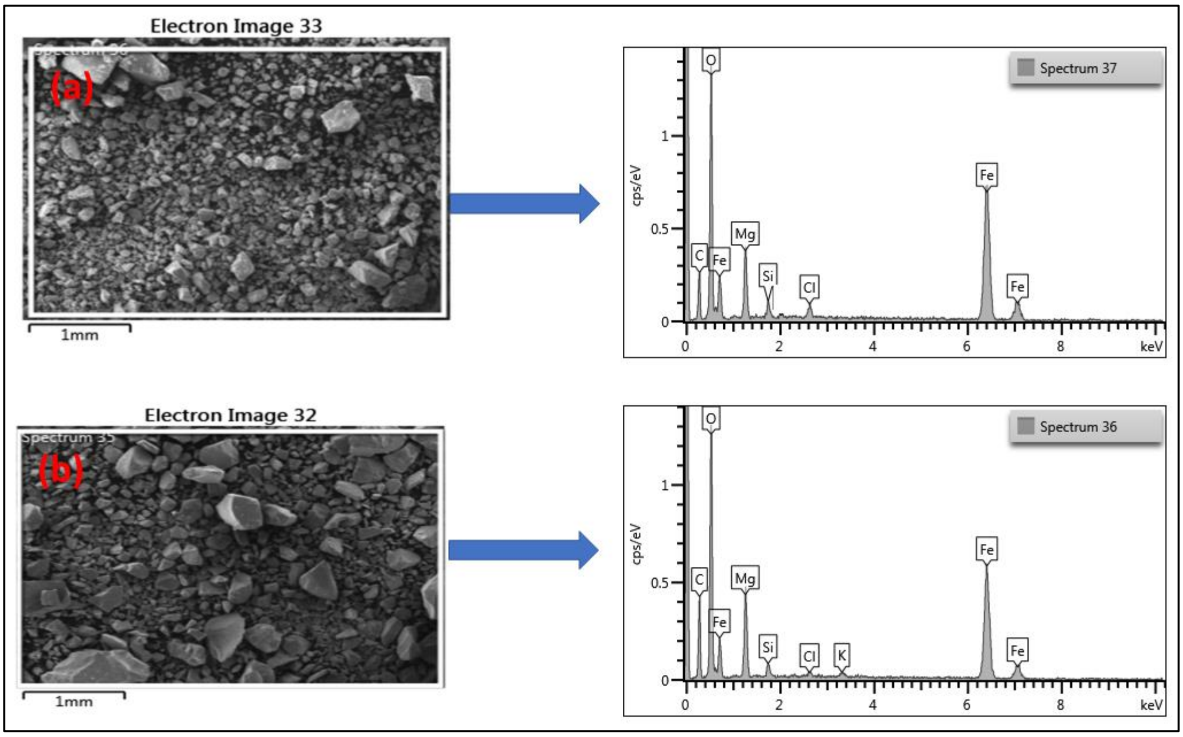

Figure S1. Electron images and elemental composition spectrums of composites by SEM-EDX technique: (**a**) $SBAC_{100}MgFe$, (**b**) $SBAC_{500}MgFe$.

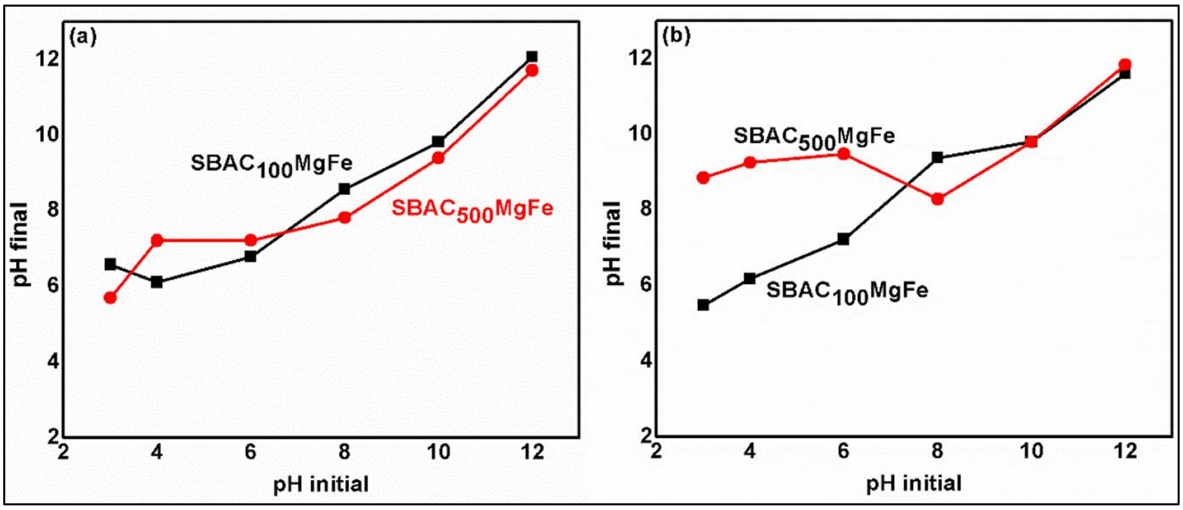

Figure S2. Equilibrium pH value of phosphate (**a**) and nitrate (**b**) after adsorption by SBAC-MgFe composites.

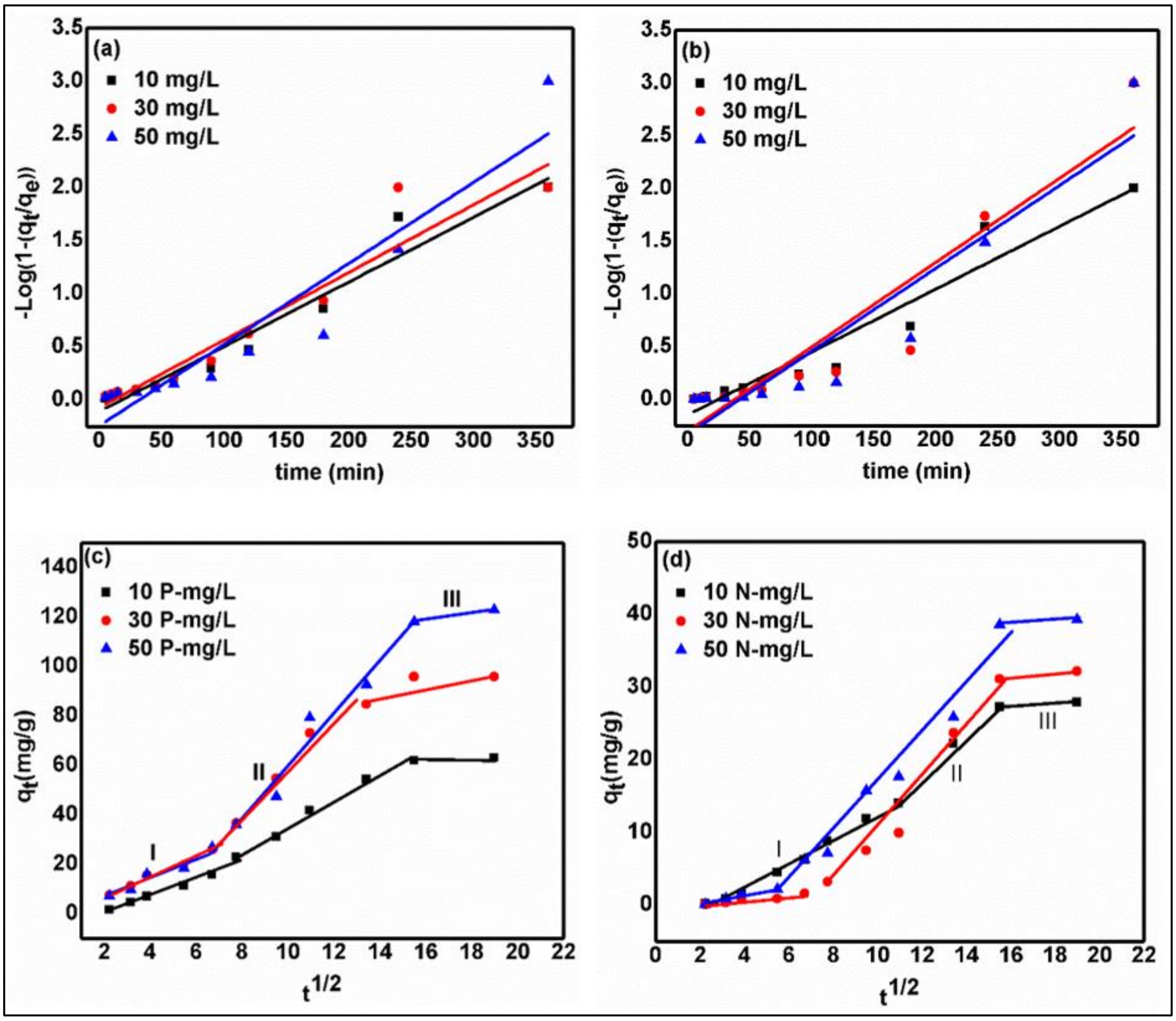

Figure S3. Film diffusion (**a**,**b**) and intraparticle diffusion (**c**,**d**) model plots for adsorption of phosphate and nitrate on $SBAC_{100}MgFe$ composite.

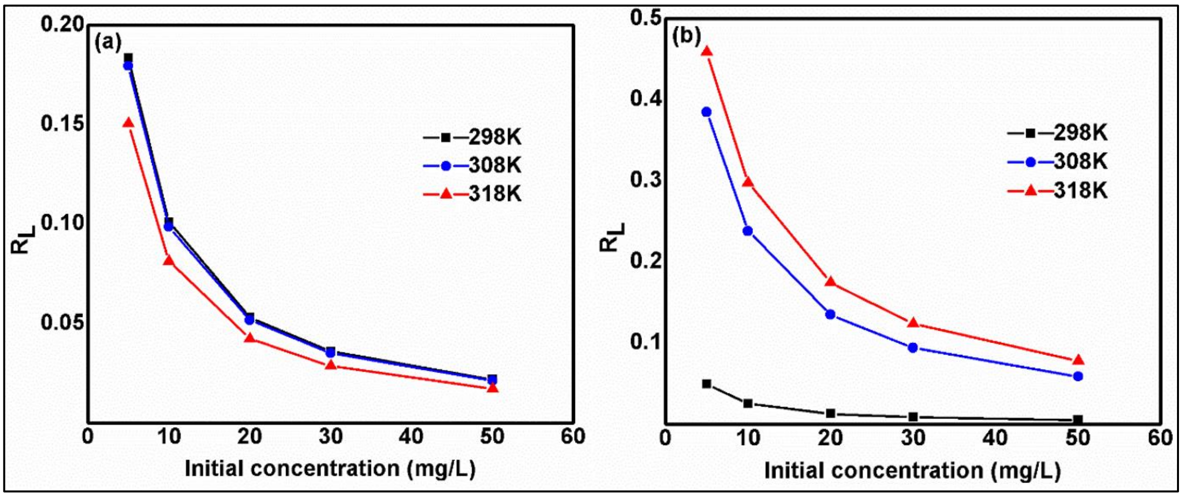

Figure S4. Plot of separation factor (R_L) versus initial concentration at three different temperature values for phosphate and nitrate adsorption on $SBAC_{100}MgFe$.

Table S1. Composition of wastewater obtained from domestic wastewater treatment plant of Imam Abdulrahman Bin Faisal University (Number of samples = 3).

Parameter	Unit	Average Value	Standard Deviation
pH	pH unit	7.23	±0.210
Temperature	°C	20.5	±0.541
Conductivity	µs/cm	677	±150
Turbidity	NTU	74.0	±5.50
Total dissolved solids	mg/L	327	±87.4
5-day biological oxygen demand (BOD_5)	mg/L	76.7	±21.0
Chemical oxygen demand (COD)	mg/L	141	±23.7
Total nitrogen	mg/L	16.0	±3.33
